# Global Transcriptional Analysis of Olfactory Genes in the Head of Pine Shoot Beetle, *Tomicus yunnanensis*

**DOI:** 10.1155/2012/491748

**Published:** 2012-06-18

**Authors:** Jia-Ying Zhu, Ning Zhao, Bin Yang

**Affiliations:** ^1^Key Laboratory of Forest Disaster Warning and Control of Yunnan Province, College of Forestry, Southwest Forestry University, Kunming 650224, China; ^2^College of Life Sciences, Southwest Forestry University, Kunming 650224, China

## Abstract

The most important proteins involved in olfaction include odorant binding protein (OBP), chemosensory protein (CSP), olfactory receptor (OR), and gustatory receptor (GR). Despite that the exhaustive genomic analysis has revealed a large number of olfactory genes in a number of model insects, it is still poorly understood for most nonmodel species. This is mostly due to the reason that the small antenna is challenging for collection. We can generally isolate one or few genes at a time by means of the traditional method. Here, we present the large-scale identifying members of the main olfactory genes from the head of *Tomicus yunnanensis* using Illumina sequencing. In a single run, we obtained over 51.8 million raw reads. These reads were assembled into 57,142 unigenes. Nearly 29,384 of them were functionally annotated in the NCBI nonredundant database. By depth analysis of the data, 11 OBPs, 8 CSPs, 18 ORs, and 8 GRs were retrieved. Sequences encoding full length proteins were further characterised for one OBP and two CSPs. The obtained olfactory genes provide a major resource in further unraveling the molecular mechanisms of *T. yunnanensis* chemoperception. This study indicates that the next generation sequencing is an attractive approach for efficient identification of olfactory genes from insects, for which the genome sequence is unavailable.

## 1. Introduction

Olfaction plays a role in almost every aspect of insect life, which represents one of the key interfaces between insects and the environment. It is used by insects to recognize a huge variety of airborne molecules for providing them with information about food, predators, and potential mates [1]. Olfaction is a complex process requiring the interaction of numerous proteins to generate a neuronal signal. The first step in the recognition of chemical signals is the odorants that enter the insects' antennae and other sensory organs via pores and travel across the hydrophobic space to the chemosensory receptors, where the response of the insect to the signal is initiated [2]. Chemoreceptor of insects is mainly formed by the olfactory receptors (ORs) and gustatory receptors (GRs), which are located in the dendritic membrane of neurons [3, 4]. As common odorants are hydrophobic molecules [5], it is difficult for them passing the aqueous barrier of the sensillar lymph surrounding the dendrites of neuronal cells [6]. During the passing process, odorants are thought to be translocated from the air to the chemoreceptors by a variety of protein mainly existing in the sensillar lymph, including odorant-binding proteins (OBPs) and chemosensory proteins (CSPs) [7–9]. Studying the genes that code for olfactory proteins could provide valuable insight into the molecular mechanisms of olfactory function.

 Since the first discovery of insect OBP in the male antennae of the giant moth *Antheraea polyphemus* [7], a large number of olfactory genes have now been identified in numerous insect species through molecular cloning, cDNA library sequencing, and genome-wide analyses [10–12]. With regard to molecular cloning, olfactory genes are assigned to divergent gene families with relatively low sequence identity (overall 20% protein sequence identity), which results in difficulty in designing appropriate primers for successful cloning [13, 14]. To some extent, cDNA library sequencing is also unable for large scale identifying olfactory genes. This is partly due to the fact that it is difficult to collect enough sensory tissues that olfactory genes restrictedly expressed for constructing the tissue targeted cDNA library because of the tiny size of sensory tissues. In addition, sequencing of limited number of randomly selected cDNA clones often have insufficient coverage of less abundant transcripts [15]. Among the available insect olfactory genes, the majority of them have been obtained by means of bioinformatic approaches based on the characteristic features of the protein families from the completed genome sequences [9, 16].

Over the past several years, the next generation sequencing technology has emerged as a cutting edge approach for high-throughput sequence determination, promptly improved the efficiency and speed of gene discover, and provided fascinating opportunities in the life sciences with dramatically reduced time, labor, and cost in nonmodel organisms [17–19]. We here took advantage of this technology to present the global transcriptome characterization of the head of *Tomicus yunnanensis*, a serious pest of *Pinus yunnanensis* occurred in southwestern China [20], with focus on comprehensively revealing the olfactory genes using Illumina sequencing. In a single run, we identified 51,822,228 raw reads, which were assembled into 57,142 unigenes. From the transcriptome database, many genes encoding major olfactory proteins were mined. These data provide an invaluable resource as a first step for grounding future studies investigating chemosensory processes. Furthermore, this study presents the simple method to efficiently uncover the divergent olfactory gene family from tiny insects.

## 2. Materials and Methods

### 2.1. Insect

Adult beetles identified based on morphological characters [21] as *T. yunnanensis* by their trunk attacking phase on *P. yunnanensis* were collected from Qujing city, Yunnan province, China.

### 2.2. RNA Extraction and Sequencing

 Head of both female and male adults were cut off and transferred to an Eppendorf tube. After grinding them under liquid nitrogen, total RNA was extracted by using TRIzol Reagent (Invitrogen) according to the manufacturer's protocol. Total RNA quantity and quality were assessed using the Agilent 2100 Bioanalyzer (Agilent technologies) with a minimum RNA integrated number value of 8. The samples for transcriptome analysis were prepared using Illumina's kit following manufacturer's recommendations. Briefly, mRNA was selected using oligo(dT) probes and then fragmented using divalent cations. cDNA was synthesized using random primers, modified and enriched for attachment to the Illumina flowcell, and then sequenced on the Illumina GA II platform. Raw data have been deposited in the NCBI Short Read Archive (SRA).

### 2.3. Bioinformatics

Prior to assembly, adaptor sequences, empty reads, and low quality sequences (reads with unknown sequences “N”) were filtered from the short raw reads. *De novo* assembly of the short reads was performed using SOAPdenovo [22] at default parameters. The generated unigenes were analyzed by searching the nonredundant (NR) database in NCBI with the BLASTx algorithm using an E-value cut-off of 10^−5^. Gene Orthology (GO) annotation was performed by Blast2GO [23] through a search of the NR database. Individual unigene with at least one match similar to olfactory genes was identified using custom databases for Blast search. ClustalX (version 1.83) [24] was used to conduct multiple sequence alignments. The presence and location of signal peptide cleavage sites in amino acid sequences was predicted by SignalP 4.0 server [25]. The software package MEGA5 [26] was used for phylogenetic analysis. Bootstrap analysis was performed using Neighbor-Joining and the Poisson correction model with 1000 replicates. Positions containing alignment gaps and missing data were eliminated with pairwise deletion.

### 2.4. PCR

To validate and extend fragments of several candidate gene sequences to encode full proteins, 5′ gene-specific primers (3′-TTCTCAATCACACACTTCAAGAAAC-5′ and 3′-TTGGTAGAATATTGGTCATCAGGTC-5′) were designed based on the sequences of Unigene41600_YNNT and Unigene26497_YNNT. They were used to clone the 5′ ends in conjunction with adapter primer provided in the SMART RACE cDNA amplification kit (Clontech). Two specific primer pairs (3′-ATGAAAACATTCGTGCTTGTTGCTT-5′ and 3′-TTACAATTTTAAACCTTCTTTTTCG-5′) were designed according to the sequence of Unigene55391_YNNT. They were employed for amplifying the open reading frame of this putative gene. cDNA template was synthesized with a SMART RACE cDNA amplification kit (Clontech) using the total RNA extracted above. PCR was performed using Advantage 2 Polymerase Mix (Clontech) according to recommended conditions and following the manufacturer's instructions.

## 3. Results and Discussion

### 3.1. Overview of the Head Transcriptome

**Figure 1:**
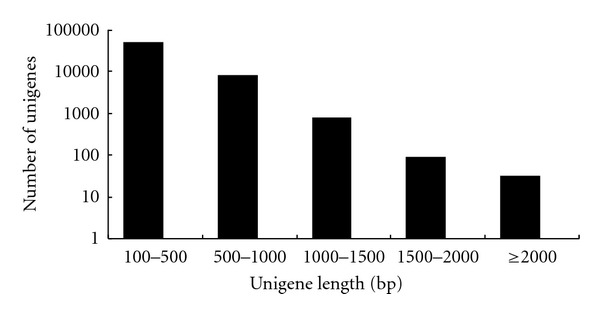
Length distribution of assembled unigenes. Note the logarithmic *y*-axis.

After cleaning and quality checks, we generated over 4,600 million bases sequence information. The total number of reads was 51,822,228 with an average length of 90 bp (Table 1). The GC percentage of the reads is 42.53%. The number is comparable with other insect and eukaryote sequencing projects that have GC content between 38.7% and 56.5% [27]. Using SOAPdenovo, these reads were assembled into 42,678 contigs, 140,311 scaffolds, and 57,142 unigenes. The average length of contigs, scaffolds and unigenes were 113 bp, 226 bp, and 355 bp, respectively. The longest unigene had 4,762 bases. The lengths of the 5,136 unigenes were ≥700 bp (Figure 1).

**Figure 2:**
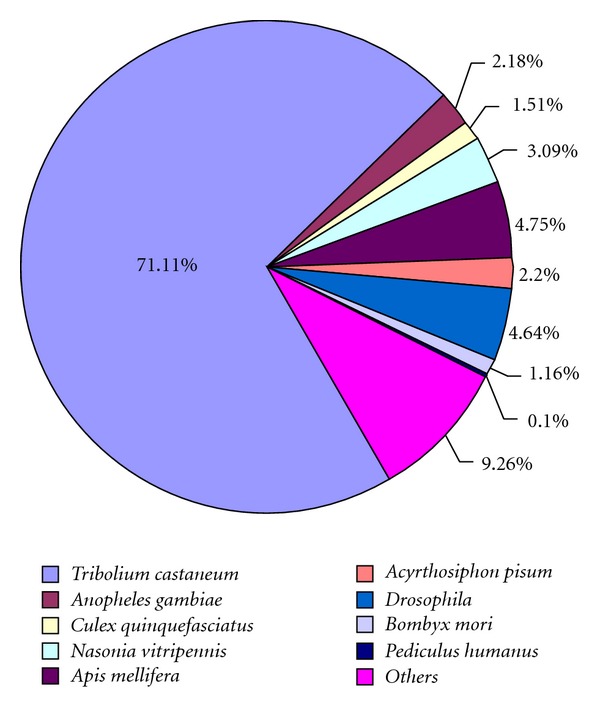
Species distribution of the top Blastx hits for unigenes from the head of *Tomicus yunnanensis* with a cutoff E-value 10^5^ to searches from the NCBI nonredundant database. The first hit of each sequence was used for analysis.

After assembly, all unigenes were aligned to NCBI NR databases with a cut-off E-value of 10^−5^ for annotation. 29,384 unigenes had at least one significant alignment to existing gene model in Blastx searches. The remaining 27,758 unigenes with no matches with any known sequences and likely represent novel. This might be due to the relatively short length of distinct gene sequences [17] and lack of genetic information in *Tomicus*. In addition, this might be resulted partly by the transcripts derived from the cDNA of untranslated regions, chimerical sequences (assemblage errors) and nonconserved areas of proteins where homology is not detected [28]. The species distribution of the best match result for each sequence is shown in (Figure 2). The *T. yunnanensis* sequences produced 20,896 hits to *Tribolium castaneum*, 1,396 hits to *Apis mellifera*, and 1,363 hits to *Drosophila*. Overall, a strong preference for matches is against *T. castaneum* genes, composing of 71.11%. It might be due to the relatively near evolutionary relationship of these two species, belonging to the same order Coleoptera, and the available complete genome sequence of *T. castaneum*.

**Figure 3:**
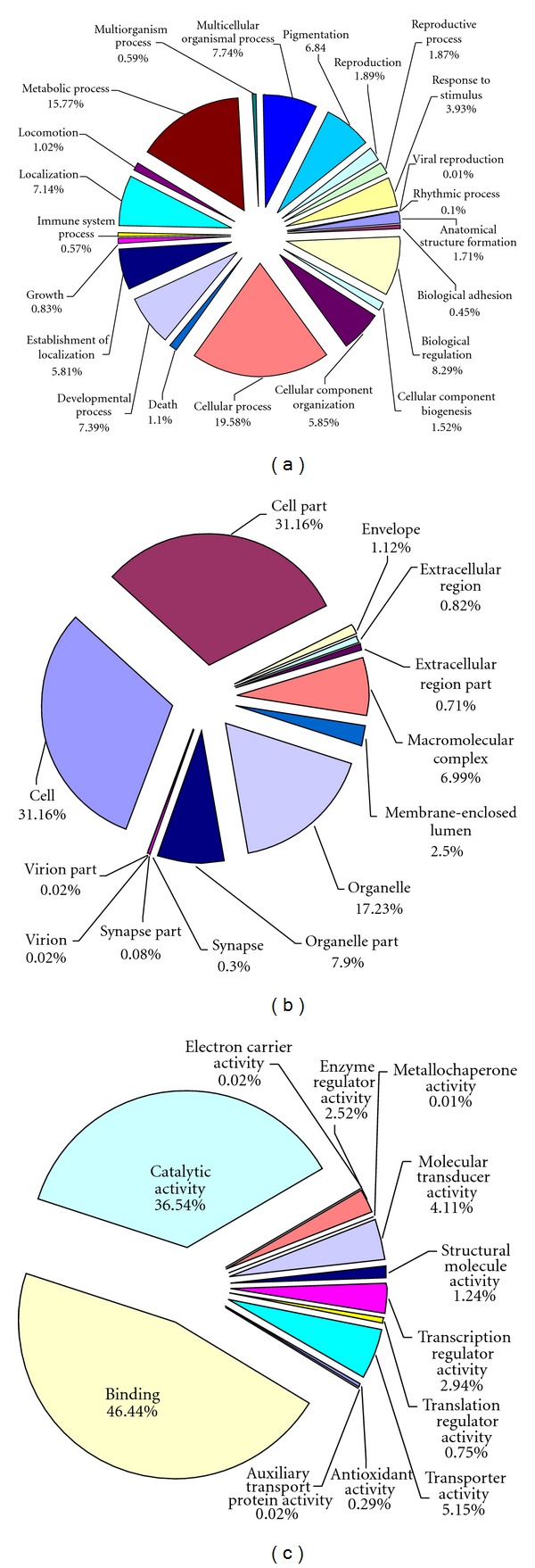
GO categories of the unigenes. (a) Molecular function GO terms, (b) biological process GO terms, and (c) cellular component GO terms. GO annotation was done with the Blast2GO tool. The data presented represent the level 2 analysis, illustrating general functional categories.

For functional comparisons, all unigenes were assigned for GO terms based on BLAST matches with sequences whose function is previously known. A total of 54,427 unigenes were able to map to GO terms. These transcripts were assigned for biological process (25,348 sequences), cellular component (17,537 sequences), and molecular function (11,542 sequences) (Figure 3). Cellular process (19.58%) and metabolic process (15.77%) were the main subcategories of biological process, indicating the important metabolic activities in *T. yunnanensis *head. Under the category of cellular component, cell (31.16%), cell part (31.16%), and organelle (17.23%) were among the most highly represented subcategories. The molecular function category was mainly comprised of proteins involved in binding (46.44%) and catalytic activities (36.54%).

### 3.2. Discovery of Genes Encoding Olfactory Proteins

Of particular interest to detect the sequences encoding olfactory proteins, the head transcriptome of *T. yunnanensis* was analyzed more in detail. Unigenes with at least one match to olfactory proteins with an E-value of 10^−5^ or lower were selected. As shown in Table 2, we identified many sequences with homology to OBP, CSP, OR, and GR. Overall, 45 potential olfactory genes out of 57,142 unigenes were identified. The proportion is similar to what was recorded in antennal transcriptome of *Manduca sexta *and in the combined EST and transcriptome data of *Solenopsis invicta* [19, 29]. Among these unigenes, 11 encoded for OBP, 8 for CSP, 18 for OR, and 8 for GR. The data in this study presents some information on the molecular basis of olfaction of a Coleoptera species besides *T. castaneum*. Analysis of fully sequenced genome has identified 49 OBPs, 20 CSPs, 299 ORs, and 220 GRs in *T. castaneum* [1, 30]. Compared with the gene number of olfactory genes reported from this genome, the current number of *T. yunnanensis* is at the lower end of the range of *T. castaneum*. Additional olfactory genes may await discovery due to their absence from the current transcriptomic dataset. The remainders not obtained from this dataset might be due to the following reasons: (1) only a single run carried out in this study, which results in the dataset that do not cover all genes expressed in the head of *T. yunnanensis*, and (2) the tissues excluded the head that some olfactory genes abundantly located are not included as the target tissue. Increasing the number of olfactory genes can discovery from transcriptome of the samples that are greatly restricted to the main olfactory organs. In addition, as the ability of Illumina sequencing is for short reads (<120 bp) [31], only one unigene is appeared to complete in all the identified putative olfactory transcripts. More candidates encoding full sequence could be determined by 454 pyrosequencing, which can generate longer sequence reads (~450 bases) than those obtained by Illumina sequencing [32, 33]. Using 454 sequencing, the majority of olfactory genes identified from the transcriptomic database of *M. sexta* antennae and *S. invicta* encoded the full length proteins [19, 29]. However, the latter technology needs much more cost.

### 3.3. Validation of Several Detected Olfactory Genes

Based on the fragments derived from the transcriptome database, primers were designed to clone the full coding sequences for one OBP (Unigene41600_YNNT) (designated as TyunOBP1) and one CSP (Unigene26497_YNNT) (designated as TyunCSP1) by RACE-PCR, and assure the acute of one CSP sequence (Unigene55391_YNNT) (designated as TyunCSP2) that appeared to be complete after raw reads assembly by RT-PCR. We have successfully performed the amplification.

**Figure 4:**
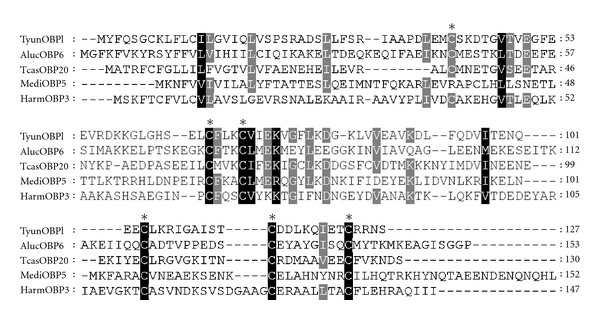
Alignment of the predicted amino acid sequence of TyunOBP1 with OBPs from other insects. The conserved cystein residues are indicated by asterisks. Black and gray indicate residues that are identical or conserved, respectively. The abbreviation and GenBank accession number for the sequences from other insect aligned are AlucOBP6, *Apolygus lucorum* (AEP95762); TcasOBP20, *Tribolium castaneum* (EFA05793); MediOBP5, *Microplitis mediator* (ABM05972), and HarmOBP3, Helicoverpa armigera (AEB54582).

**Figure 5:**
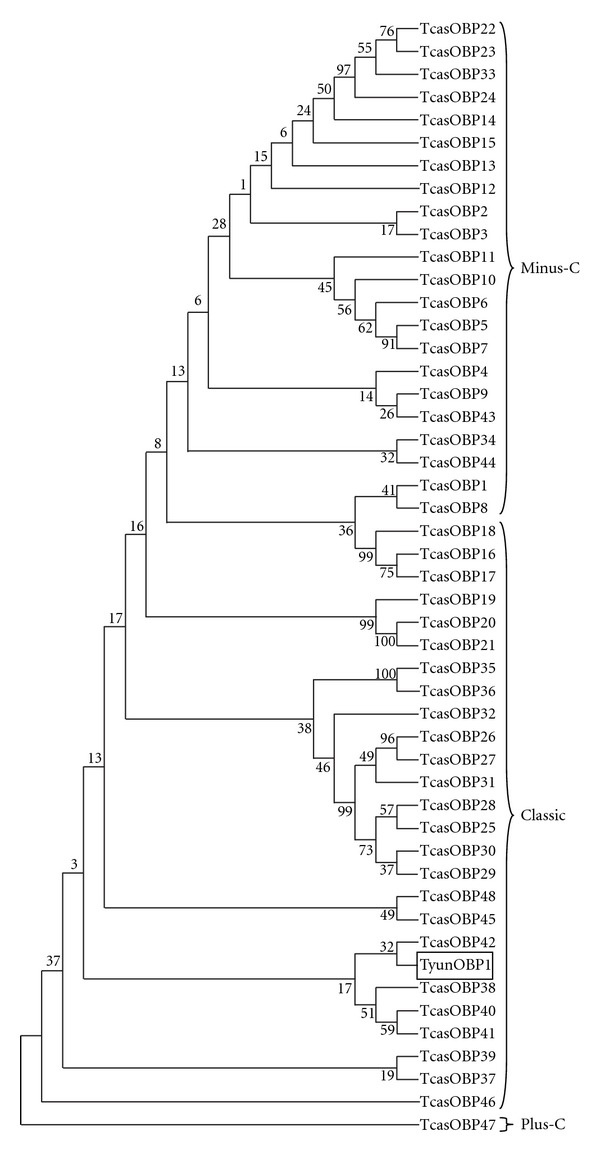
Neighbour-joining phylogenetic analysis of OBPs from *Tomicus yunnanensis* (Tyun) and *Tribolium castaneum* (Tcas). Sequences from *T. yunnanensis* are marked by a box. Numbers at nodes are bootstrap values shown as percentages. The *T. castaneum* OBP gene family was classified according to Sánchez-Gracia et al. [16].

TyunOBP1 contained a 384 bp open reading frame (ORF) for a polypeptide of 128 amino acids. It was with a molecular mass of 14,15 Da and an isoelectric point (pI) of 5.15. The deduced protein sequence was in accordance with other OBPs, generally composed of subunits of about 14 kDa as small hydrophilic proteins with acidic isoelectric points [34]. Insect OBPs have also been classified into long chain (~160 aa), medium chain (~120 aa), and short chain (~110 aa) classes, which relates to their potential structure and function [35]. According to this hypothesis, TyunOBP1 belongs to the medium chain class. A signal peptide is a common characteristic of OBPs [36]. Similar to that of other insects, the initial 25 amino acids of TyunOBP1 were predicted as a signal peptide. In addition, OBPs are characterized by conserved cysteines that are believed to form three disulfide bridges, and the hydrophobic domains [37]. Multiple amino acid sequence alignment revealed that the six cysteine residues are highly conserved in TyunOBP1 (Figure 4). Moreover, it indicated that TyunOBP1 displayed low similarity to that of other insects, which are typically around 20% or below, and the lengths of the N- and C-termini of OBPs are highly divergent. This highly diverse insect gene family is divided into nine major subclasses: Classic, Minus-C, Plus C, Dimer, PBP/GOBP, ABPI, ABPII, CRLBP, and D7 [16, 38]. Among the currently available genomes, the dipterans consistently have large expansions of these genes [39], and the body louse,* Pediculus humanus*, appears to possess the smallest set of OBPs [40]. A phylogenetic tree was constructed using the amino acid sequences of TyunOBP1 and *T. castaneum* OBP (TcasOBP) (Figure 5). According to the phylogenetic tree and the features of OBP subclasses, TyunOBP1 is near to TcasOBP42, belonging to the Classic clad.

**Figure 6:**
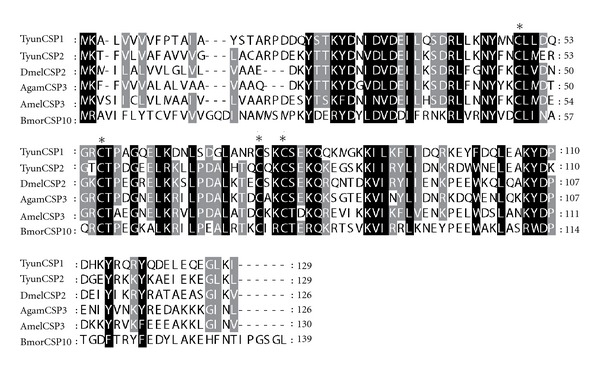
Alignment of the predicted amino acid sequence of TyunCSP1 and TyunCSP2 with CSPs from other insects. The conserved cystein residues are indicated by asterisks. Black and gray indicate residues that are identical or conserved, respectively. The abbreviation and GenBank accession number for the sequences from other insect aligned are DmelCSP2, *Drosophila melanogaster* (NP_524966); AgamCSP3, *Anopheles gambiae* (EAA12338); BmorCSP10, *Bombyx mori* (NP_001037069), and AmelCSP3, *Apis mellifera* (NP_001011583).

**Figure 7:**
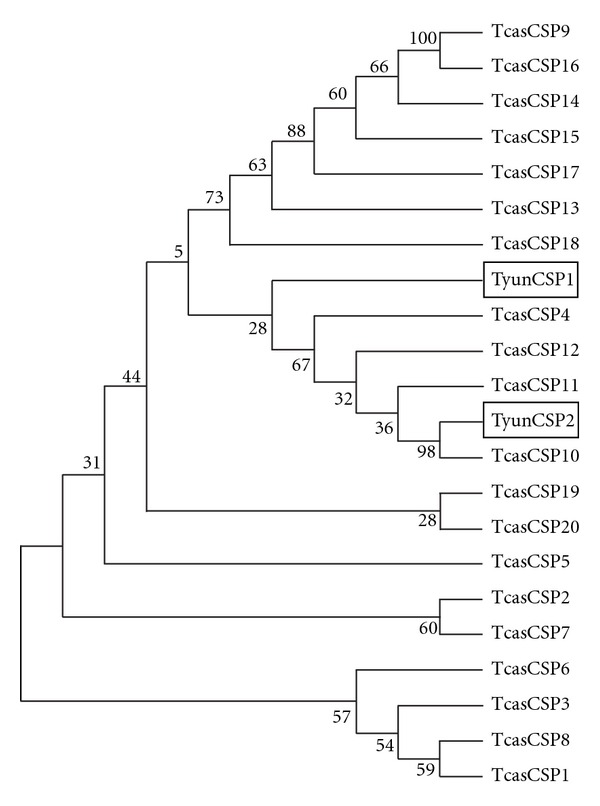
Neighbour-joining phylogenetic analysis of CSPs from *Tomicus yunnanensis* (Tyun) and *Tribolium castaneum* (Tcas). Sequences from *T. yunnanensis* are marked by a box. Numbers at nodes are bootstrap values shown as percentages.

The ORF for TyunCSP1 and TyunCSP2 were 390 bp. Both of them consisted of 130 amino acids. TyunCSP1 was with a calculated molecular weight of 15.11 kDa and pI of 5.10, whereas TyunCSP2 was with a molecular mass of 15.17 Da and a pI of 8.02. All CSPs appear to have a hydrophobic N-terminus of about 16–20 amino acids that are predicted to encode a signal peptide [41]. The deduced amino acid sequences for both proteins possessed a putative signal sequence of 18 amino acids. A typical feature for CSP is the remarkably conserved 4-cysteine motif that forms disulphide bridges in folded proteins [42]. A comparison between the sequences of TyunCSP1, TyunCSP2, and those of other insects clearly showed that a conserved four-cysteine signature is present at the characteristic positions within the CSP family (Figure 6). CSPs are more conserved than OBPs across evolution, with about a 50% identity even between members of phylogenetically distant species [43]. These two TyunCSPs shared 54% identity, but were with 26–58% identity to CSPs from other species used in the multiple alignments. To characterize the molecular evolution relationships between the CSPs of *T. yunnanensis* and those of others insects in Coleoptera, we constructed a phylogenetic tree including 2 CSPs of *T. yunnanensis* identified in this study, and 20 CSPs of *T. castaneum* (Figure 7). The tree showed that five proposed groups of CSPs were resolved. TyunCSP1, TyunCSP2, and most of the CSPs of *T. castaneum* were grouped into the largest distinctive phylogenetic clade.

## 4. Conclusion

Historically, identification of a comprehensive list of candidate olfaction genes for understanding the molecular mechanisms of olfaction has been mostly limited by the difficulty in collecting antenna because it is small and encased in a hard cuticle, and successful cloning as the olfactory gene families are divergent [44]. Next generation short-read DNA sequencing has made it possible to explore genome-level questions in nonmodel organisms, regardless of their phylogenetic proximity to model species [45, 46]. This study produced a large amount of transcriptome data from *T. yunnanensis *head by Illumina sequencing. An extensive list of candidate genes involved in olfactory signal transduction has been generated from this valuable data platform. This is an important resource for further study of the chemical communication between* T. yunnanensis* and the environment. This study demonstrated that the Illumina sequencing technology could be applied as a particularly rapid, cost-effective, and fruitful approach to accelerate our understanding of the molecular basis of chemosensory pathways in nonmodel insects that have no previous genome data.

## Figures and Tables

**Table 1:** Summary of Illumina sequencing of *Tomicus yunnanensis* head transcripts.

	Reads	Contigs	Scaffolds	Unigenes
Number of sequences	51,822,228	671,467	140,311	57,142
Mean length (bp)	90	113	226	355
Total length (bp)	4,664,000,520	75,947,228	31,643,690	20,291,442

**Table 2:** Putatively identified olfactory genes in *Tomicus yunnanensis. *

Gene family	Unigene ID	Length (bp)	First hit	E_value	Blast annotation/Organism
Ordorant binding protein	Unigene13495_YNNT	279	EFA12066	6.00E-06	Odorant binding protein 15 [*Tribolium castaneum*]
	Unigene14057_YNNT	415	EFA07430	1.00E-06	Odorant binding protein (subfamily minus C) C04 [*Tribolium castaneum*]
	Unigene2588_YNNT	282	EEZ97740	2.00E-07	Odorant binding protein C12 [*Tribolium castaneum*]
	Unigene41600_YNNT	256	EFA05793	5.00E-06	Odorant binding protein 20 [*Tribolium castaneum*]
	Unigene41993_YNNT	258	EFA10713	5.00E-19	Odorant binding protein 09 [*Tribolium castaneum*]
	Unigene44812_YNNT	277	EFA07430	3.00E-06	Odorant binding protein (subfamily minus C) C04 [*Tribolium castaneum*]
	Unigene4914_YNNT	231	EFA10803	2.00E-07	Odorant binding protein 23 [*Tribolium castaneum*]
	Unigene54663_YNNT	466	EFA07430	2.00E-13	Odorant binding protein (subfamily minus C) C04 [*Tribolium castaneum*]
	Unigene6724_YNNT	361	EFA01425	5.00E-10	Odorant binding protein C20 [*Tribolium castaneum*]
	Unigene7827_YNNT	311	AAD31883	4.00E-09	Odorant binding protein RpalOBP2^′^ [*Rhynchophorus palmarum*]
	Unigene9635_YNNT	315	EFA05695	7E-28	Odorant binding protein 11 [*Tribolium castaneum*]

Chemosensory protein	Unigene10309_YNNT	360	NP_001039280	2.00E-15	Chemosensory protein 12 [*Tribolium castaneum*]
	Unigene13359_YNNT	219	NP_001039274	8.00E-17	Chemosensory protein 20 [*Tribolium castaneum*]
	Unigene19380_YNNT	498	BAF91720	1.00E-22	Chemosensory protein [*Papilio xuthus*]
	Unigene24674_YNNT	250	NP_001039290	8.00E-11	Chemosensory protein 8 [*Tribolium castaneum*]
	Unigene24747_YNNT	1891	NP_001039288	6.00E-26	Chemosensory protein 6 [*Tribolium castaneum*]
	Unigene2482_YNNT	235	NP_001039288	2.00E-12	Chemosensory protein 6 [*Tribolium castaneum*]
	Unigene26497_YNNT	356	NP_001039278	2.00E-34	Chemosensory protein 10 [*Tribolium castaneum*]
	Unigene55391_YNNT	512	NP_001039278	1E-60	Chemosensory protein 10 [*Tribolium castaneum*]

Olfactory receptor	Unigene10465_YNNT	223	XP_001507235	7.00E-07	Predicted: similar to olfactory receptor MOR18-3 [*Ornithorhynchus anatinus*]
	Unigene1058_YNNT	236	XP_001512662	1.00E-17	Predicted: similar to olfactory receptor OR19-14, partial [*Ornithorhynchus anatinus*]
	Unigene16616_YNNT	426	XP_001511079	4.00E-13	Predicted: similar to olfactory receptor Olr4 [*Ornithorhynchus anatinus*]
	Unigene17391_YNNT	522	XP_002932395	2.00E-06	Predicted: olfactory receptor 10A4-like [*Xenopus (Silurana) tropicalis*]
	Unigene22269_YNNT	303	XP_001512662	2.00E-20	Predicted: similar to olfactory receptor OR19-14, partial [*Ornithorhynchus anatinus*]
	Unigene25110_YNNT	837	XP_001507235	3.00E-43	Predicted: similar to olfactory receptor MOR18-3 [*Ornithorhynchus anatinus*]
	Unigene25351_YNNT	285	XP_001368212	1.00E-12	Predicted: similar to C-family odorant receptor OLFCT1 [*Monodelphis domestica*]
	Unigene27701_YNNT	391	EFA09172	2.00E-08	Odorant receptor 12 [*Tribolium castaneum*]
	Unigene30603_YNNT	205	XP_001513226	8.00E-19	Predicted: similar to olfactory receptor MOR253-9 [*Ornithorhynchus anatinus*]
	Unigene30802_YNNT	206	CAM84014	1.00E-27	Olfactory receptor 16 [*Tribolium castaneum*]
	Unigene3238_YNNT	419	XP_001516663	2.00E-46	Predicted: similar to olfactory receptor MOR218-3 [*Ornithorhynchus anatinus*]
	Unigene45804_YNNT	286	CAM84014	2.00E-40	Olfactory receptor 16 [*Tribolium castaneum*]
	Unigene47392_YNNT	300	XP_001516663	3.00E-30	Predicted: similar to olfactory receptor MOR218-3 [*Ornithorhynchus anatinus*]
	Unigene51999_YNNT	372	CAM84014	2.00E-30	Olfactory receptor 16 [*Tribolium castaneum*]
	Unigene53119_YNNT	403	BAG12817	2.00E-11	Olfactory receptor-like receptor [*Bombyx mori*]
	Unigene53416_YNNT	412	XP_002932395	3.00E-08	Predicted: olfactory receptor 10A4-like [*Xenopus (Silurana) tropicalis*]
	Unigene54915_YNNT	479	ACD40044	3.00E-66	Odorant receptor [*Phyllotreta striolata*]
	Unigene9988_YNNT	617	XP_002932775	0.0000006	Predicted: olfactory receptor 11L1-like [*Xenopus (Silurana) tropicalis*]

Gustatory receptor	Unigene13393_YNNT	259	NP_001138958	2.00E-18	Gustatory receptor Gr85 [*Tribolium castaneum*]
	Unigene14213_YNNT	261	CAL23158	6.00E-06	Gustatory receptor candidate 25 [*Tribolium castaneum*]
	Unigene1791_YNNT	463	NP_001138958	3.00E-25	Gustatory receptor Gr85 [*Tribolium castaneum*]
	Unigene24414_YNNT	906	NP_001138948	2.00E-69	Gustatory receptor Gr83 [*Tribolium castaneum*]
	Unigene31152_YNNT	207	EFA13587	2.00E-09	Gustatory receptor 21 [*Tribolium castaneum*]
	Unigene35383_YNNT	223	EFA02924	2.00E-18	Gustatory receptor 2 [*Tribolium castaneum*]
	Unigene4039_YNNT	493	NP_001138958	2.00E-29	Gustatory receptor Gr85 [*Tribolium castaneum*]
	Unigene44392_YNNT	274	NP_001138958	2.00E-20	Gustatory receptor Gr85 [*Tribolium castaneum*]
